# Detection of intraneural needle-placement with multiple frequency bioimpedance monitoring: a novel method

**DOI:** 10.1007/s10877-015-9698-3

**Published:** 2015-04-23

**Authors:** Håvard Kalvøy, Axel R. Sauter

**Affiliations:** Department of Clinical and Biomedical Engineering, Oslo University Hospital Rikshospitalet, Oslo, Norway; Division of Emergencies and Critical Care, Department of Anaesthesiology, Oslo University Hospital Rikshospitalet, Oslo, Norway

**Keywords:** Bioimpedance, Monitoring, Needle, Nerve, Nerve stimulation, Regional anaesthesia

## Abstract

Electrical impedance measurements have been used to detect intraneural needle placement, but there is still a lack of precision with this method. The purpose of the study was to develop a method for the discrimination of nerve tissue from other tissue types based on multiple frequency impedance measurements. Impedance measurements with 25 different frequencies between 1.26 and 398 kHz were obtained in eight pigs while placing the tip of a stimulation needle within the sciatic nerve and in other tissues. Various impedance variables and measurement frequencies were tested for tissue discrimination. Best tissue discrimination was obtained by using three different impedance parameters with optimal measurement frequencies: *Modulus* (126 kHz), *Phase angle* (40 kHz) and the *Delta* of the phase angle (between 126 and 158 kHz). These variables were combined in a *Compound variable C.* The area under the curve in a receiver operating characteristic was consecutively increased for the *Modulus* (78 %), *Phase angle* (86 %), *Delta* of the phase angle (94 %), and the *Compound variable C* (97 %), indicating highest specificity and sensitivity for *C*. An algorithm based on *C* was implemented in a real-time feasibility test and used in an additional test animal to demonstrate our new method. Discrimination between nerve tissue and other tissue types was improved by combining several impedance variables at multiple measurement frequencies.

**Electronic supplementary material:**

The online version of this article (doi:10.1007/s10877-015-9698-3) contains supplementary material, which is available to authorized users.

## Introduction

Nerve injuries related to peripheral nerve blocks can be caused by toxicity of the injected solution or by mechanical nerve damage. In the worst cases, nerve damage can lead to persistent motor or sensory impairment and debilitating neuropathic pain [1, 2]. Thus, it is highly important to avoid such iatrogenic injuries. Penetration of a nerve alone does not necessarily lead to lasting damage unless local anaesthetic is injected within the nerve fascicle [3]. Hence, if intraneural needle placement is identified in time, the needle could be withdrawn and nerve injury can be avoided. Ultrasound guidance [4], electrical nerve stimulation [5–7], and injection pressure measurements [8, 9] are used to reduce the risk of intraneural needle placement and injection. A combination of these methods is recommended to reduce the risk of intraneural needle placement and injection when peripheral nerve blocks are performed [10]. However, the reliability of these methods to reduce the incidence of nerve injuries has not been demonstrated [11].

Impedance is a measure of the opposition of the flow of alternating current (AC) similar to the resistance of a conductor to direct current (DC). Impedance is given as a complex number that can be described as a vector in a complex plane defined by the *Modulus* and the *Phase Angle*, unlike resistance in a DC circuit that has only a magnitude which is expressed by a single value [12]. The *Modulus* is the ratio of the voltage amplitude to the current amplitude and is given in ohms. The *Modulus* defines the length of a vector in the complex impedance plane. The *Phase Angle* reflects the phase shift by which the current delays behind the applied voltage and is given in degrees. The *Phase angle* defines the direction of the vector in the impedance plane. All biological impedance variables depend on the frequency of the applied measurement signal.

Electrical bioimpedance in a needle-electrode circuit has been measured in an animal model and in clinical studies to detect placement of the block needle within a nerve [13, 14]. The bioimpedance was measured as an absolute value obtained with a square pulse from an electrical nerve stimulator. Advancing the needle through tissue types with different electrical conductivity can give a rise or fall in the measured impedance. Until now, sufficient discrimination of a nerve from other tissue types has not been obtained using such absolute impedance measurements.

In the present study, bioimpedance measurements were made with multiple frequencies as described in a previous publication [15]. The complex impedance data set was examined for tissue specific patterns. Our hypothesis was that the specific curve shapes obtained by plotting the impedance variables as a function of the frequencies could be used as a specific “fingerprint” to reliably identify and discriminate nerve tissue from other tissue types.

## Materials and methods

After institutional animal care committee approval (ID 5143) nine pigs were supplied for this study by the Center for Comparative Medicine (Oslo University Hospital, Rikshospitalet, Oslo, Norway). General anesthesia with endotracheal intubation was administered with fentanyl and isoflurane after premedication with ketamin, azaperone, and atropin.

We used an ATL HDI 5000 ultrasound unit with a L12-5 transducer (ATL, Bothell, Washington, USA) to visualize the left sciatic nerve in an oblique axis below the biceps femoris muscle. The unilateral setup was due to a consecutive study that was scheduled for the same test animals after bioimpedance measurements were performed. A 100 mm, 21-gauge insulated needle with a 30° bevel (Stimuplex^**®**^ A; B. Braun, Melsungen, Germany) was used without fluid priming. The needle was advanced from anterolateral to posteromedial under in-plane ultrasound guidance until the needle-tip was positioned within the epineurium of the sciatic nerve.

A nerve stimulator (Stimuplex^**®**^ HNS 12; B. Braun, Melsungen, Germany) was connected to the needle and a gel reference electrode (Blue Sensor, Q-00-A, Ag/AgCl, Ambu Medicotest A/S, Denmark) on the skin. The current threshold needed to obtain a neuromuscular response was established starting at 0 mA and gradually increasing the current (2 Hz frequency, 0.1 ms impulse width). The nerve stimulator was removed and a Solartron complex impedance measurement system (SI 1260 and SI 1294, Solartron Group PLC, Hampshire, UK) was connected for 3-electrode impedance measurements. The needle was connected as the measuring electrode and two skin electrodes were attached to the skin of the lower abdomen for use as indifferent electrodes. A 50 mV excitation signal was driven by the Solartron 1294 between the needle and one of the skin electrodes, and the resultant potential was measured between the needle and the other skin electrode. This 3-electrode setup, and the large differences in electrode area between the needle and the skin electrodes, ensured a unipolar impedance measurement totally dominated by the needle and the tissue adjacent to its tip [16]. Impedance as function of frequency was obtained by sweeping the excitation frequency in 25 logarithmically distributed steps from 1.26 to 398 kHz.

**Fig. 1:**
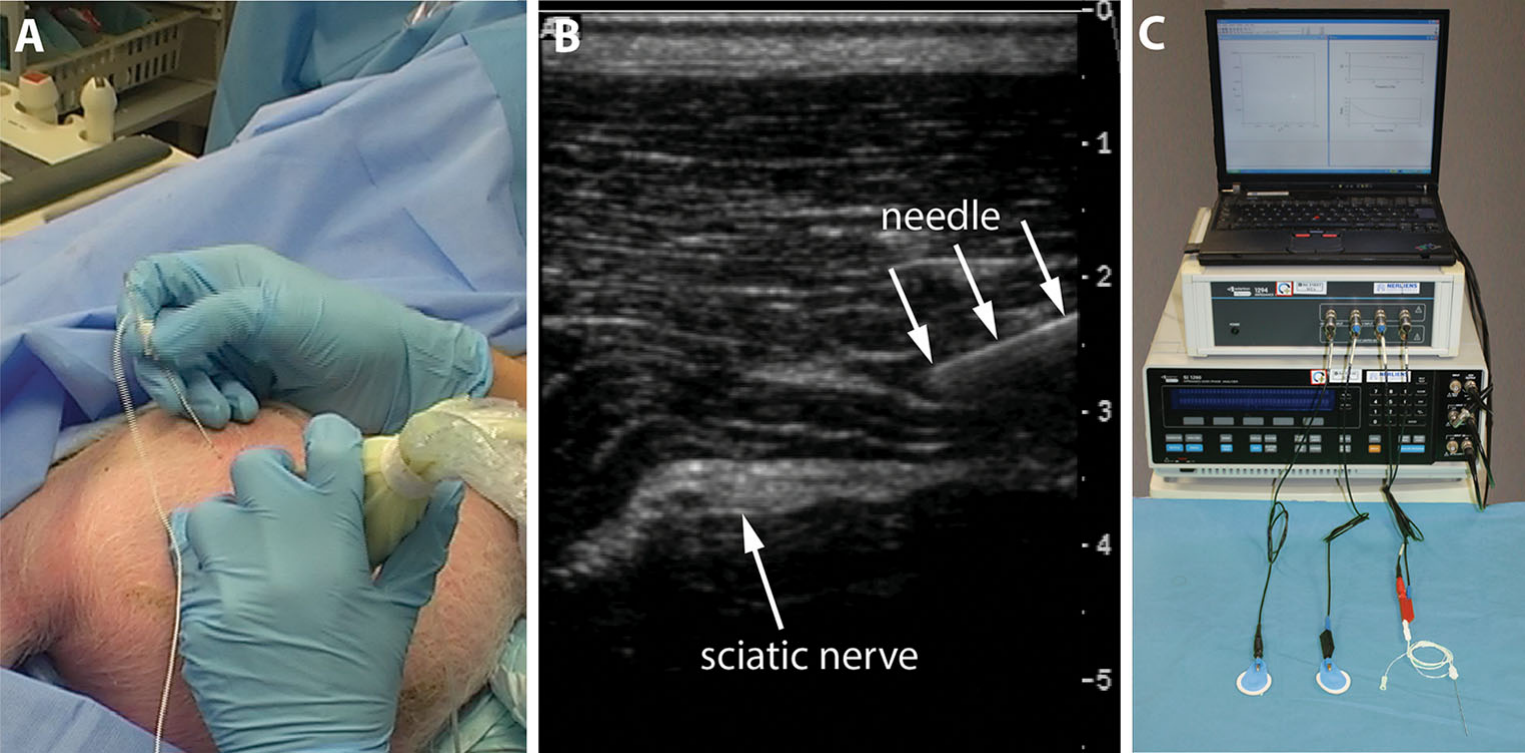
Study setup: The tip of a stimulation needle was placed under ultrasound guidance within the sciatic nerve and other tissue types (**a** and **b**). An impedance measurement system (Solartron 1260 and SI 1294) was connected for 3-electrode impedance measurements (**c**). Impedance as function of frequency was obtained by sweeping the excitation frequency in 25 logarithmically distributed steps from 1.26 to 398 kHz

The needle-tip was then placed in a second, third and fourth position within the epineurium of the nerve and bioimpedance measurements were repeated. The needle was withdrawn from the nerve and placed in four different paraneural positions, defined by visualisation of the needle-tip between the hypoechoic tissue of the biceps femoris muscle and the hyperechoic sciatic nerve and surrounding connective tissue layers. Thereafter the needle was placed in four positions within the biceps femoris muscle and in four positions within the subcutaneous fat. Impedance as function of frequency was measured for each needle position. Figure 1 gives an illustration of the study setup.

### Processing of data and statistics

All the statistical analyses were conducted in R (Team 2010) for MAC [17]. The impedance values (*Modulus* and *Phase angle*) were plotted graphically for visual interpretation to assemble characteristic properties from the measured data set for defined frequency ranges. Descriptive statistics including principal component analysis (PCA) were used to illustrate variances and compare the measurements in order to identify tissue specific differences.

The information in the PCA plots was used to derive two new variables: The *Delta* of the phase angle was defined as the difference in measured phase angles between two consecutive measurement frequencies; The *Compound variable C* was derived by manual identification of tissue specific patterns in the PCA plots. One-Way ANOVA was used to evaluate the mean differences between tissue types versus intraneural (Holm–Sidak for assumed normal distribution, and Kruskal–Walis if normal distribution could not be assumed from a Shapiro–Wilk normality test).

A receiver operating characteristic curve (ROC) was a final test and comparison of the tissue discrimination variables, where the area under the curve (AUC) was used as an indicator of how well a variable can distinguish between nerve tissue and other tissue types. For the *Modulus*, *Phase angle,* and *Delta* we defined the mean value in intraneural tissue as the starting point with zero positive classification (“true positive” = 0 and “false positive” = 0, in the plot). The cut off range was then extended by moving the upper and lower cut off values in steps of 5 %, until all the values in the total data set were included at 100 %. *C* was normalized to zero at the intraneural mean value. Hence, zero was used as the starting point for *C*. The cut of range for *C* was extended by increasing the value by adding 5 %.

To demonstrate the feasibility of the method in a real-time clinical device an algorithm based on the best variables was used in a generic impedance measurement (PXI platform, National Instruments Corportion, Austin, TX and Solartron SI 1294). The feasibility test was performed in the ninth test animal.

## Results

The sciatic nerve and surrounding anatomy was identified unilaterally in eight 3 months old pigs (24–30 kg, 4 female and 4 male). Current thresholds for a distal muscle response ranged from 0.04 to 0.28 mA when the needle was placed within the sciatic nerve. 128 datasets from bioimpedance measurements in a frequency range from 1.26 to 398 kHz were obtained when the needle-tips were either placed intraepineurally, paraneurally, within muscle tissue, or in subcutaneous fat. 17 of these datasets were excluded as missing data because of contact failure in the needle connection lead or disturbance of the measurement setup.

**Fig. 2:**
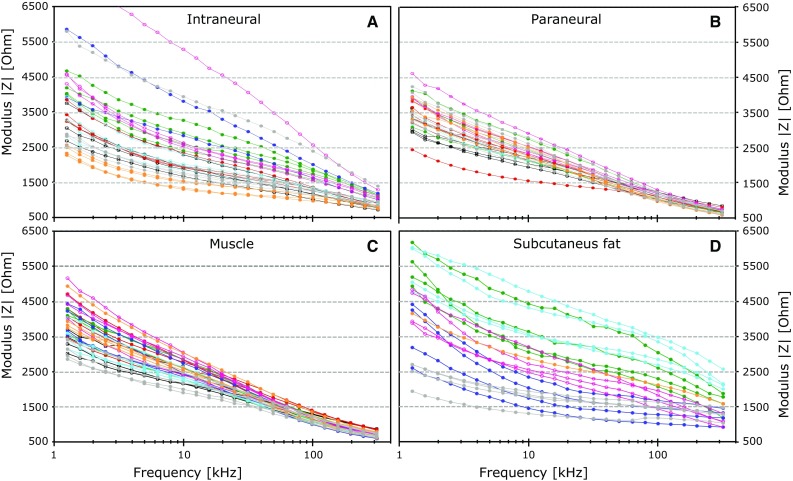
Measurements of the impedance *Modulus* in a frequency range from 1.26 to 398 kHz when the needle tip was positioned in intraneural tissue (**a**), paraneural tissue (**b**), muscle (**c**) and subcutaneous fat (**d**). *Each color* represents repeated measurements from one test animal. The *y* axis gives the *Modulus* in ohms; the *x* axis gives the measurement frequencies in Hertz on a logarithmic scale

**Fig. 3:**
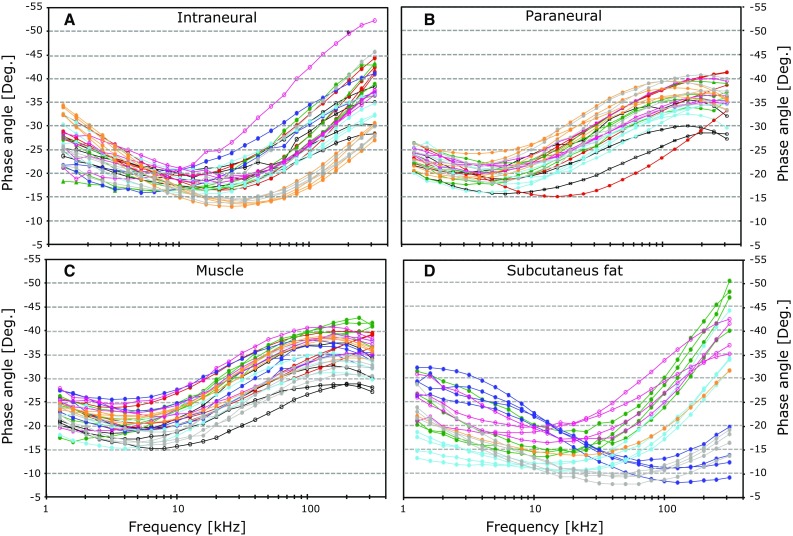
Measurements of the impedance *Phase angel* in a frequency range from 1.26 to 398 kHz when the needle tip was positioned in intraneural tissue (**a**), paraneural tissue (**b**), muscle (**c**) and subcutaneous fat (**d**). *Each color* represents repeated measurements from one test animal. The *y* axis gives the *Phase angle* in degrees; the *x* axis gives the measurement frequencies in Hertz on a logarithmic scale

Figures 2 and 3 show how the measured bioimpedance changes as a function of the applied frequencies. With visual assessment, a noticeable decrease in the modulus above 20 kHz can be seen for muscle and paraneural tissue in Fig. 2b, c. This corresponds to a distinct increase in the phase angle in Fig. 3b, c that reaches its peak value between 100 and 200 kHz. A similar pattern was found for the intraneural measurement, but in a higher frequency range. The decease in the modulus and the corresponding increase in phase angle are found above 60 kHz (Figs. 2a, 3a).

**Fig. 4:**
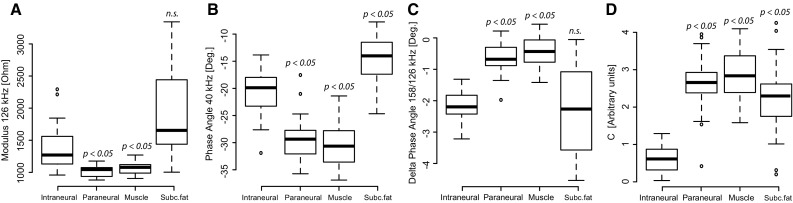
Parameters used to discriminate intraneural needle positions from positions in other tissue types. Statistical analysis (PCA) showed best tissue discrimination at 126 kHz for the *Modulus* (**a**), at 40 kHz for the *Phase angle* (**b**), at a *Delta phase angle* between 126 and 158 kHz (**c**). Tissue discrimination was further improved by combining these measurements in a *Compound variable* *C* (**d**). The *box plot* denotes median, quartile, range and outliers. The *quoted p values* relates to the statistical differences of means versus intraneural needle positions. Not significant differences are denoted *n.s*

PCA plots of the data obtained with each of the 25 measurement frequencies showed best discrimination between intraneural tissue and the other tissue types at 126 kHz for the modulus (Fig. 4a) and at 40 kHz for the phase angle (Fig. 4b). For the *Delt*a phase angle (defined as the difference in measured phase angles between two consecutive measurement frequencies) best results were obtained between 126 and 158 kHz (Fig. 4c). The tissue discrimination could further be improved when combining *Modulus*, *Phase angel* and *Delta* (at the given frequencies) in a *Compound variable C*, according to the following equation:

$$ C = \sqrt {\left( {\frac{{ - M_{{126{\text{kHz}}}} - 73.8 \times P_{{40{\text{kHz}}}} - 65.9}}{562}} \right)^{2} + \left( {\frac{{P_{{158{\text{kHz}}}} - P_{{126{\text{kHz}}}} + 2.27}}{0.95}} \right)^{2} } $$

where M is the *Modulus* and P is the *Phase angle* at the frequencies given by the subscripts (Fig. 4d).

**Fig. 5:**
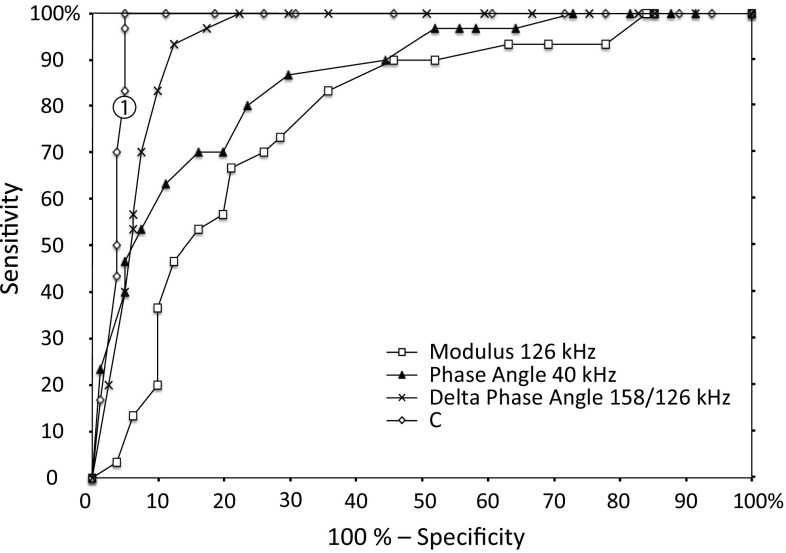
Receiver operating characteristic curve (ROC) for the four parameters used to discriminate intraneural needle placement from other tissue types. *True positive* values (sensitivity) and *False positive* values (${\text{100 \%}} - {\text{specificity}}$) are plotted while increasing cutoff in steps of 5 % from the lowest to the highest values. The area under the curve (AUC) is consecutively increasing for the *Modulus* (78 %), *Phase angle* (86 %), *Delta* (94 %) and the *Compound variable C* (97 %). Best specificity and sensitivity can be obtained by using the compound parameter *C*. The predefined cutoff value *C* equals *1* that was used for our prototype (See video 1, electronic supplementary material) is labeled with (*1*)

The four variables used to discriminate intraneural needle placement from other tissue types were plotted in a ROC curve (Fig. 5). The AUC is consecutively increased for the *Modulus* (78 %), *Phase angle* (86 %), *Delta* (94 %), and *C* (97 %). This indicates highest specificity and sensitivity for *C*.

The real-time feasibility test to discriminate intraneural needle placement from other tissue types is documented in a video sequence (See video 1, electronic supplementary material). The setup was programmed with an indicator colour panel that turned from green to red when the *Compound variable* was below a predefined cutoff value of *C* = 1. Based on the ROC (Fig. 5) this should give a sensitivity of 80 % and a specificity of 96 % for the discrimination for intraneural tissue.

## Discussion

A novel algorithm for bioimpedance measurements to detect nerve tissue, and discriminate it from surrounding tissue types, was developed by analysing a complex impedance dataset based on multiple measurement frequencies. Several impedance variables were combined in the *Compound variable C* to optimise tissue discrimination.

The specific curve shapes obtained by plotting the impedance variables as a function of the frequencies (the tissue specific beta-dispersion [15, 18]) were used as a “fingerprint” to identify and discriminate nerve tissue from other tissue types. When analysing various impedance variables, best tissue discrimination was found for the impedance *Modulus* at a measurement frequency of 126 kHz, for the *Phase angle* at 40 kHz, and for the *Delta* of the phase angle between 126 kHz and 158 kHz. Compared with the *Modulus,* discrimination was improved with the *Phase angle*, and even better when using *Delta* (at the optimal measurement frequencies).

The absolute values for the *Modulus* and *Phase angle* measured at a single frequency are highly dependent on the measurement setup and the electrodes used [19]. The *Delta* is mainly based on the specific curve shape and is less vulnerable to inconstancies. Combining these measurements in the variable *C* made the discrimination even more robust.

Our study has several limitations. First, our measurements were obtained in an exploratory animal study and refer only to the sciatic nerve in pigs. Comparisons by Gabriel (28) shows that the impedance properties of animal tissues are comparable to human tissues, and that the variations within a species may well exceed variations between species. The differences they found between human and animal species were not systematic. At the present time, comparable data for peripheral nerves are not available.

Different peripheral nerves might have different conductive properties. It must be emphasised that the sciatic nerve is formed by to independent nerve structures, a tibial and a peroneal component that are surrounded by a distinct connective tissue layer [20]. Marked differences in neural architecture and the size of surrounding adipose tissue compartments have been demonstrated between proximal and distal parts of peripheral nerves in humans [21]. The proportion of connective tissue in the course of a peripheral nerve may range from 30 to 75 % [22]. Thus altered frequency dependent impedance patterns might not only be found between different nerves, but also within the different measurement positions in a single nerve. It might therefore be necessary to modify our present algorithm when data from humans and from other nerves are available. Another possibility would be to use multiple specific algorithms depending on the anatomical site. Further, clinical studies must confirm that our results are applicable in humans.

The stimulation needle was placed in multiple positions within the sciatic nerve. Repeated needle advancements might change the conductive properties of the nerve. This was not the case in our study. No statistical correlation for repeated measurements was found in our data.

In our study the needles were not primed with fluid. For the Stimuplex^**®**^ A needles, most of the electrical contact between tissue and the needle is obtained on the bevel surface. When the needle is filled with conductive solutions the distal part of the needle cavity will contribute to current conduction. However, body fluids will also pass into the needle during block procedures. This can alter the impedance in the cavity for both primed and unprimed needles. Ideally, block needles could be optimized for impedance measurements by isolating its cavity surface (in addition to the outer surface) to reduce variations in the electrode area caused by different fluid contents.

Ultrasound is known as an observer-dependent method; the identification of anatomical structures and estimation of the position of the needle-tip have a subjective component. To insure true intraneural needle positions we chose a large nerve that was easy to identify and to access. With an in-plane needle approach we aimed for a central position of the needle-tip in the middle of the nerve. When placing the needle in the paraneural tissue, indentation of the nerve wall was avoided. Intraneural injections to confirm needle positions by a typical spread of the injectate [23] were not performed because we did not want to affect the native electrical properties of the tissue or alter impedance in consecutive measurements by performing injections. Hence, we cannot exclude errors despite subjectively adequate ultrasound visualisation and the low current thresholds that were found for the intraneural needle positions.

Even though highly clinically relevant, we could not specify whether the needle was placed within the neural fascicles or in the surrounding perineurium. Neither did we investigate needle positions in transition from the extra- to the intra-epineural space. To identify needle position within smaller structures or compartments in our porcine model, nerves might have to be surgically exposed. However, we don’t expect reliable and representative bioimpedance patterns after removing the paraneural tissue from the nerve as the current path and the electrical field would be changed or impeded [12]. The lack of differentiation between intraneural structures is a major limitation of our study.

Our measurements show distinct patterns for subcutaneous fat, muscle, and intraneural tissue. The results obtained from the paraneural needle positions were almost identical to the measurements in muscle, as can be seen in Figs. 2, 3 and 4. This is probably caused by the conductive properties from muscle tissue surrounding the sciatic nerve, that dominate the bioimpedance measurements in the paraneural positions. In our porcine study, it was difficult to distinguish between epineurium and surrounding connective tissue layers. Hence, our porcine model is not appropriate to investigate close needle-to-nerve contact. Detection of nerve contact at an early stage before the needle is penetrating the epineurium is clinically important and must be addressed in future studies.

Temperature and fluid status in the tissue can be confounding variables in bioimpedance measurements. Temperature coefficients for body tissues are unlikely to exceed 1–2 % per °C, and are most significant at low frequencies [24]. Fluid changes of 1.5 L in healthy volunteers are shown to give 4–5 % alteration in single frequency bioimpedance measurements [25]. These changes are relatively low compared to the natural variation of impedance in biological tissue types. Hence, within a clinical range of temperature and fluid status, we expect these factors to have minimal confounding for our multiple frequency algorithm.

In the present study, a controlled potential of 50 mV was used to ensure a low current density. This should give a linear relationship between current and potential. High current density, on the other hand, could lead to a nonlinear behaviour of the measurement setup and irreproducible results [12, 26].

Whereas our measurements were based on defined sine wave frequencies, others have used a square pulse signal (obtained from an electrical nerve stimulator) for bioimpedance measurements. Tsui et al. [14] performed bioimpedance measurements in pigs and found a higher impedance intraneurally compared with the extraneural muscle tissue. Bardou et al. [13] measured bioimpedance in 140 peripheral nerve blocks. When nerve puncture was suspected in 21 of these cases, a relative increase of impedance was typically found. However, an alteration of absolute impedance might also be found when the needle tip is moved into other tissue types with a low electrical conductivity. When comparing needle positions in muscle tissue with a needle placement in fat or connective tissue in a previous study, a 50 % increment of the measured impedance was found [27].

According to Fourier’s theory, a square pulse represents multiple frequencies. [12, 28] Impulses with a short duration can mainly be derived from high frequent sine waves; in this case the electrical current can relatively easily pass through the capacitive tissue membranes. Impulses, with a long duration, as used for electrical nerve stimulation, imply more low-frequency components; this causes biological membranes to act like a fully charged capacitor that will oppose the current flow and cause an increase in the measured bioimpedance [18]. To obtain reproducible results, both the electrical current and impulse duration must be kept constant when bioimpedance measurements are performed with square pulse signals as in the study by Bardou et al. [13]. However, our present study showed that the AUC of 67 % obtained by Bardou et al. could be improved to 97 % by introducing controlled frequency measurement.

The method seems feasible for use in clinical practice to perform continuous bioimpedance monitoring during peripheral nerve block performance. An algorithm based on *C* was implemented in a generic impedance measurement device and used in an additional animal test to confirm our findings (See video 1, electronic supplementary material).

We anticipate that our method for tissue discrimination might be implemented in a nerve stimulator. Multiple frequency bioimpedance measurements could be made in the pauses between stimulation pulses. Previous studies have shown specific bioimpedance patterns for a variety of organs and tissue types [29]. Bioimpedance has also been used for the localization of blood vessels [30]. A clinical measurement device could combine multiple algorithms; not only to indicate an accidental intraepineural needle position, but also to display other tissue types, or for instance needle-placement within a blood vessel. A clinical device for the identification of nerve tissue on the other hand might not only be used for PNB. The field of application could as well include procedures like radio- and cryoablation to obtain a reliable identification of the target nerves.

Electrical impedance measurements are used in numerous clinical devices [12]. The measurement potential in our study is only 0.05 V, which is very small compared to commercially available nerve stimulators, that are capable to deliver pulses up to 90 V. Hence, an impedance measurement device based on our algorithm could pass approval formalities for clinical use in human subjects as long as the equipment is manufactured according to electrical safety regulations.

## Conclusion

We have developed a novel algorithm based on complex impedance measurements at multiple frequencies, and defined sine wave excitation, which is able to discriminate needle positions in nerve tissue from other tissue types. Clinical studies in humans are needed to confirm our results.

## Electronic supplementary material

Video 1 Real-time feasibility test to discriminate intraneural needle placement from other tissue types: The setup was programmed with an indicator colour panel that turned from green to red when the *Compound variable* was below a predefined cutoff value of *C* = 1. This should give a sensitivity of 80% and a specificity of 96% for the discrimination for intraneural tissue (MP4 9577 kb)
